# Removal of a 10-kb *Gret1* transposon from *VvMybA1* of *Vitis vinifera* cv. Chardonnay

**DOI:** 10.1093/hr/uhac201

**Published:** 2022-09-06

**Authors:** Yingzhen Yang, John Ke, Xiaoyan Han, Wegi A Wuddineh, Guo-qing Song, Gan-Yuan Zhong

**Affiliations:** USDA-Agricultural Research Service Grape Genetics Research Unit, Geneva, NY 14456, USA; USDA-Agricultural Research Service Grape Genetics Research Unit, Geneva, NY 14456, USA; Department of Horticulture, Michigan State University, East Lansing, MI 48823, USA; USDA-Agricultural Research Service Grape Genetics Research Unit, Geneva, NY 14456, USA; Department of Horticulture, Michigan State University, East Lansing, MI 48823, USA; USDA-Agricultural Research Service Grape Genetics Research Unit, Geneva, NY 14456, USA

## Abstract

Many white grape cultivars have a nonfunctional *VvMybA1* gene due to the presence of a 10-kb *Gret1* transposon in its promoter. In this study, we successfully demonstrated removal of the 10-kb *Gret1* transposon and functional restoration of a *VvMybA1* allele in *Vitis vinifera* cv. Chardonnay through transgenic expression of Cas9 and two gRNAs simultaneously targeting two junction sequences between *Gret1* LTRs and *VvMybA1*. We generated 67 and 24 Cas9-positive vines via *Agrobacterium*-mediated and biolistic bombardment transformation, respectively. While the editing efficiencies were as high as 17% for the 5′ target site and 65% for the 3′ target site, simultaneous editing of both 5′ and 3′ target sites resulting in the removal of *Gret1* transposon from the *VvMybA1* promoter was 0.5% or less in most transgenic calli, suggesting that these calli had very limited numbers of cells with the *Gret1* removed. Nevertheless, two bombardment-transformed vines, which shared the same unique editing features and were likely derived from a singly edited event, were found to have the *Gret1* successfully edited out from one of their two *VvMybA1* alleles. The edited allele was functionally restored based on the detection of its expression and a positive coloring assay result in leaves. Precise removal of more than a 10-kb DNA fragment from a gene locus in grape broadens the possibilities of using gene editing technologies to modify various trait genes in grapes and other plants.

## Introduction

Berry color is an important fruit quality trait in grapes (*Vitis*) and it is largely influenced by the content of anthocyanins in berry skin. Besides their direct role in the appearance of fresh grape berries, anthocyanins and their derived compounds also contribute to many sensory attributes, nutritional and health benefits in both table grapes and processed grape products. Anthocyanin content in berry skin is controlled by the color locus containing 4 *MYB*-type transcription factor genes, *VvMybA1-VvMybA4*, located in a span of ~140 kb region on chromosome 2 [1–4]. *VvMybA1* and *VvMybA2* play critical roles in determining anthocyanin content in grape berries while *VvMybA3* and *VvMybA4* encode truncated MybA proteins and are not functional [1, 5–7]. Lack of anthocyanin pigments in most white grape cultivars is attributed to the simultaneous loss of *VvMybA1* gene expression due to insertion of a ~ 10-kb *Gret1* transposon in the promoter and loss-of-function mutations in the *VvMybA2* coding region resulting from a non-conserved point mutation and a 2-bp frame-shift deletion [1, 6–8].

*VvMybA1* with *Gret1* insertion is present not only in white grape cultivars of *V. vinifera* but also widely in its progenitor species *V. sylvestris* [9]. Function of a mutated *VvMybA1* gene can be restored by excision of *Gret1* through homologous recombination between long terminal regions (LTRs) flanking the *Gret1* as first confirmed in the bud mutants Ruby Okuyama and Flame Muscat [8]*.* The presence of several similar *VvMybA* genes in cluster may also result in illegitimate recombination between these genes leading to creation of new *VvMybA* genes and phenotypes [5, 10]. For instance, an illegitimate homologous recombination between *VvMybA1* and *VvMybA3* was reported to result in a hybrid *MybA* gene with restored gene function and berry color [11]. Cultivars containing large deletion involving the removal of both *VvMybA1* and *VvMybA2* [4, 6, 12–14] and large chromosome re-arrangements in the color locus [15] have also been reported.

Gene editing with CRISPR-Cas9 in grapes has been successfully applied for single target mutagenesis, including *L-idonate dehydrogenase* in “Chardonnay” [16], *Phytoene Desaturase* (*PDS*) in “Muscat” [17] and “Chardonnay” [18], *WRKY52* gene in “Thompson Seedless” [19], and *MybA7* and *Trans-Acting Small-interfering locus 4b* (*TAS4b*) in rootstock 101–14 [20]. Intended large deletion of genomic fragments through CRISPR-Cas9 editing has also been reported in the animal system, such as deletion of 10 kb – 0.5 Mb in mice [21–23], 1 Mb deletion in Zebra fish [24] as well as various sizes of deletion in human cell lines [25]. Similar studies were also reported in the plant system, including deletion of a few hundred bps to a few kbs in soybean [26], 115–245 kbs in rice [27], 50–100 bps in tomato [28], and ~ 1 kb in Arabidopsis [29]. Preferential formation of small deletions was observed in the deletion editing studies in both Arabidopsis and *Nicotiana benthamiana*, suggesting that the efficiencies of deletion were reduced with the increase in the deletion size [30]. So far, CRISPR/Cas9 mediated large deletions have not been reported in grape.

There are many economically important white grape cultivars and restoration of berry color in these cultivars may provide additional choices for grape industry and consumers. In this study, we report successful removal of the 10-kb *Gret1* transposon from a *VvMybA1* allele in *V. vinifera* cv. Chardonnay through CRISPR/Cas9 editing of two target sites flanking the transposon. This work demonstrates significant technical potential for simultaneous editing of two target sites more than 10 kbs apart and provides important insights into modification of transposon-affected genes in grapes and other crops, especially those that are perennial and clonally propagated.

## Results

### Generation and identification of transgenic lines

The MG1 construct was used to transform Chardonnay embryogenic callus by either agrobacterium-mediated or biolistic transformation and the resulting transgenic calli were maintained on hygromycin selection medium for at least 6 months before molecular analysis and regeneration of stable transgenic plants. We regenerated a total of 80 and 106 hygromycin-resistant vines from the callus transformed *via Agrobacterium* and biolistic bombardment method, respectively. Sixty seven of the 80 transgenic vines obtained from *Agrobacterium*-mediated transformation (83.8%) and 24 of the 106 vines from biolistic transformation (22.3%) were tested positive for the presence of *Cas9* by genomic PCR. This difference between the two transformation methods might be due to the disruptive nature of bombardment to the integrity of the plasmid DNA/T-DNA of the transformation construct [36]. We focused our further analyses on the 91 Cas9-positive transgenic lines.

### Detection of double editing events in *VvMybA1* locus

**Figure 1:**
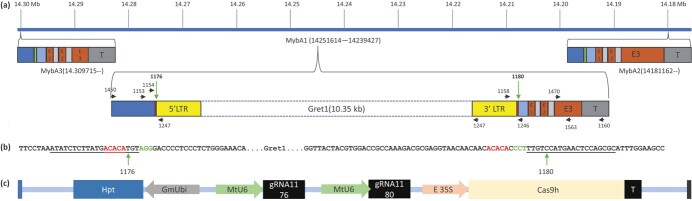
Schematic Illustration of the complex *VvMybA* loci in the distal region of chromosome 2 and the molecular designs for editing out *Gret1* from *VyMybA1* in Chardonnay. (a) Schematic diagram of the complex *VvMybA* loci in Chardonnay. *VvMybA* contains three highly homologous loci, *MybA3*, *MybA1* and *MybA2*, which can be distinguished from one another based on SNP and structural variations. T stands for terminator, E for exons, and LTR for long terminal repeat. Primers and their positions are indicated by arrows and numbers, and Cas9 target sites marked by green arrows. Primer 1160 is specific to *VvMybA1*. The *Gret1* transposon (unsolid box in *VvMybA1*) is about 10.35 kb and bordered by two LTRs (5′ LTR and 3′ LTR, in yellow) and further flanked by a target sequence duplication (TSD) on each side (red). 1450/1160 or 1153/1160 prime pairs were used for long amplicon PCRs across the *Gret1* transposon region in *VvMybA1*. The primer pairs of 1154/1247 and 1158/1246 were used to amplify the Cas9 target region between the *MybA1* promoter and the 5′ LTR and between the *MybA1* promoter and 3′ LTR, respectively. The *MybA1*-specifc primer pairs of 1470/1563 in exon 3 were used in RT-PCR for detection of *MybA1* gene expression. (b) *Gret1* and promoter junction sequences in the *VvMybA1* locus. Cas9 target sequences were underlined. PAM (NGG) sequences were in green. TSDs were in red. Ideal cutting positions (3 bp upstream of PAM site) for target sites 1176 and 1180 were pointed by green arrows. (c) Schematic illustration of the *VvMybA1* editing construct MG1. Left border: blue strip; right border: black strip; Hpt: hygromycin resistance gene; GmUbi: ubiquitin promoter from soybean; MtU6:U6 small RNA promoter from Medicago; gRNA1176 and gRNA1180: small guiding RNAs targeting *MybA1*-*Gret1* junctions; E35S: enhanced 35S promoter; Cas9h: human codon-optimized *Cas9* gene; T: terminator.

**Figure 2:**
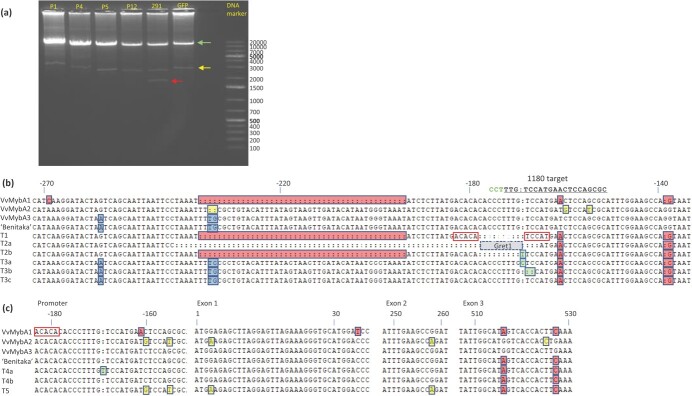
Editing-induced DNA structural and sequence variants at the *VvMybA1* locus. (a) Long amplicon genomic PCRs of transgenic callus samples. P1, P4, P5 were callus samples transformed with CRISPR-Cas9 constructs not relevant to this study and served as control. P12 was a callus sample transformed with a *VvMybA1* editing construct not reported in this study. 291 was a callus sample transformed with MG1. GFP was transgenic callus carrying a 35S:GFP construct and served as additional control. The primer pair 1153/1160 (Fig. 1A) was used for the genomic PCR. A major band (green arrow) of ~12.1 kb was present in all samples and supposedly contained unedited *VvMybA1* containing the *Gret1* transposon. A 2.6-kb band (yellow arrow) of unknow origin was also present in all samples. A ~ 1.8-kb band (red arrow) was specific to the callus sample transformed with MG1. (b, c) Structural variants resulted from gene editing of the *VvMybA1* locus. Sequences of 5 large structural variants (T1-T5), based on cloning and sequencing of the ~1.8 kb band in (a), were aligned with the reference sequences of *VvMybA1*, *VvMybA2* and *VvMybA3* from “Pinot Noir”*.* Diagnostic indels and SNPs for differentiating *VvMybA1*, *VvMybA2* and *VvMYbA3* were shaded in red, yellow, and blue color, respectively. The sequence of the known mutant “Benitaka” was also included for comparison. (b) Structural variants of T1, T2 and T3. T1 is a result of perfect editing and the most common editing variant (Tables 1 and S3). “ACACA” and “TCCAT” which are at the 3 bp upstream of the 1176 and 1180 target PAM sites, respectively, were marked with red rectangles. The *Gret1* transposon (a grey box with dotted outline) was located between “ACACA” and “TCCAT”. T2 is also a type of double editing but with extra indels (See Tables T1 and S3 for more editing variants). T3 has gene editing footprints (marked by green box: insertion of “C” in T3a or “::” 2 bp deletion in T3b) and appears to be a hybrid sequence between *VvMybA3* and *VvMybA1*. The position was labeled as the distance from the “ATG” start codon. (c) Structural variants of T4 and T5. T4 is similar to the *VvMybA* allele in “Benitaka”. Both T4a and T4b also appear to be a hybrid between *VvMybA3* and *VvMybA1* as what was observed in T3, but the hybrid sequence involved in both promoter and coding regions. T4a had a footprint of gene editing with an insertion of “T” at the 1180 target site. T5 is rare and seems to be a hybrid between *VvMybA2* and *VvMybA1.* The three *MybA* genes share identical sequence (~258 bps) between position 258 (part of exon 2) and position 516 (beginning of the exon 3).

The *Gret1* transposon could be removed from *VvMybA1* by simultaneously editing two Cas9 target sites flanking the transposon. We referred such editing events as double editing in this report. To detect double editing events, we conducted genomic PCRs of transformed callus using a Long Amp Taq DNA Polymerase amplification system with one primer located in the *VvMybA1* promoter and the other primer located in the *VvMybA1* terminator (Fig. 1a). While the primers 1153 or 1450 could bind to the promoter regions of *VvMybA1*, *VvMybA3* and/or *VvMybA2* due to the high homology among the three *VvMybA* genes, the primer 1160 only anneals to the *VvMybA1* terminator region (Fig. 1a and Table S1). We expected to generate a PCR band of ~12.1 kb with the primer pair 1153/1160 or ~ 12.5 kb with 1450/1160 (Fig. 1a) from a *Gret1*-containing *VvMybA1* allele. If the 10-kb *Gret1* was deleted, a much smaller band should be amplified. We conducted PCR on several transgenic callus samples of different sources using the 1153/1160 primer pair (Fig. 2a). A faint PCR band of ~1.8 kb was detected in the callus sample 291 which was transformed with MG1, but not in other callus samples (Fig. 2a). Sample P12 was a callus sample transformed with a construct also for removal of the *Gret1* with a different gRNA at the 3’LTR target, but the construct was not further tested due to its much less editing efficiency as shown in the *in vitro* test of target efficiencies (Fig. S1). The expected 1.8-kb band was not observed in the P12 sample. The other three samples were non-*MybA1*-related transgenic callus samples. All samples had a PCR band of ~2.6 kb as well as the expected ~12.1 kb band from un-edited *MybA1* alleles (Fig. 2a). The 2.6 kb band was cloned and sequenced (Fig. S2) from multiple samples and likely resulted from direct recombination between 5′ LTRs and 3′ LTRs flanking *Gret1* in *VvMybA1*, as observed in the *VvMybA1b* allele in “Ruby Okuyama” [8]. Based on the relative band intensity (Fig. 2a), such naturally-occurring recombination took place very infrequently.

**Figure 3:**
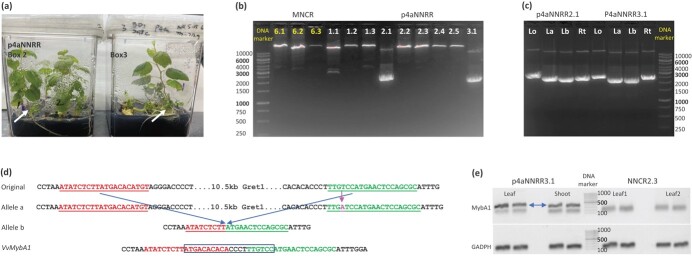
Identification and characterization of two transgenic vines with *Gret1* removed from one of their *VvMybA1* alleles. (a) These transgenic plants were regenerated from bombardment-transformed callus. Two transgenic plants with the *Gret1* removed from one of their *VvMybA1* alleles (arrows) were identified. (b) LongAmp genomic PCR results amplified with the primer pair 1450/1160 from 12 bombardment-transformed plants, including 6 from Fig. 3a. Plants p4aNNRR2.1 and p4aNNRR3.1 had a strong PCR band indicating the presence of a *Gret1*-free *VvMybA1* allele. (c) LongAmp genomic PCR results with multiple samples from p4aNNRR2.1 and p4aNNRR3.1: one root sample (Rt) and three leaf samples which included one (Lo) sampled at an early developmental stage (as in B) and two additional samples (La and Lb) sampled 3 weeks later. The sizes of ~12.5 Kb and ~ 2.2 Kb bands appeared varying among samples in Figures 3b and 3c. The size variation was artifacts likely caused by the SYBR Green since the sequence identifies of clones from the~2.2 Kb were the same. (d) Schematic and sequence illustration of two major *VvMybA1* alleles detected in p4aNNRR2.1 and p4aNNRR3.1. Target sequences for gRNA1176 and gRNA1180 were colored in red and green, respectively. An un-edited *VvMybA1* in Chardonnay was listed on the top. One allele (a) is almost identical to the original *VvMybA1*, but with a nucleotide “A” (colored purple) inserted at 3 bps upstream of the PAM sequence at the 3′ target site. The second allele (b) is a *VvMybA1* allele with the *Gret1* edited out and the two target sites joined together with a deletion of extra 11 bps. The 20 bp difference between Allele b and the wild-type *VvMybA1* is boxed. (e) Detection of *VvMybA1* expression in p4aNNRR3.1 vine. NNCR2.3 was used as the negative control. It was regenerated from the same batch of transgenic 2018 Chardonnay callus but the *Gret1* was not edited out. 45 cycles were used for the RT-PCR reactions with *MybA1*-specific primers (see Methods for detail). A house-keeping gene of *GADPH* was used as control. Both leaf tissue and multiple re-generated shoots from p4aNNRR3.1 were tested and multiple reactions were carried out for each sample. Two reactions for two samples were presented. The p4aNNRR3.1-unique bands (indicated by blue arrow) were gel-purifed, cloned, sequenced and confirmed to be *MybA1*.

Cloning and sequencing the ~1.8-kb band from the callus sample 291 identified five main types (T1-T5) of *VvMybA* editing variants with the *Gret1* transposon removed (Fig. 2b and 2c). T1 was a result of perfect double editing with breaking and rejoining points at the predicted position of 3 bps upstream of both PAM sites, while T2 had additional deletions or insertions in the resulting target sequences (Fig. 2b). T3 and T4 were likely hybrids between *VvMybA3* and *VvMybA1*, but their occurrences were relatively infrequent. T3 had *VvMybA3* features before the transposon junction and *VvMybA1* features after the 1180 target site (Fig. 2b). T4 had the *VvMybA1* feature noticeable after exon 2, but *VvMybA3* features before exon 3 (Fig. 2c). T4 structure is very similar to what was reported in the sport color mutant “Benitaka” [11]. Some T4 clones carried gene editing footprints at the 1180 target site (Fig. 2c). T5 was rare and appeared to be a hybrid between *VvMybA2* and *VvMybA1* with *VvMybA1* features also noticeable after exon 2 (Fig. 2c).

Since primer 1153 can potentially bind to all three *VvMybA* genes, we could identify double editing variants of *VvMybA1* with the *Gret1* edited out (Types 1 and 2) as well as hybrid *VvMybA* variants between *VvMybA3* and *VvMybA1* (Types 3 and 4) or between *VvMybA2* and *VvMybA1* (Type 5). Subsequently, we used the primer pair 1450 (bind to *VvMybA1* and *VvMybA3*) and 1160 for screening transgenic callus and all 91 Cas9-postive vines for the evidence of double editing. Almost all vine samples, with only two exceptions, produced no or only a very faint PCR band of ~2.2 kb presumably resulted from double editing (Fig. 3b), suggesting that double editing resulting in the removal of *Gret1* took place only in a small portion of the cells in these vines.

Based on the relative intensity of the ~2.2 kb PCR band, we estimated that transgenic vines had about 0.01% - 0.5% of the *VvMybA1* alleles with the *Gret1* removed (Fig. S3). Two plants, p4aNNRR2.1 and p4aNNRR3.1 which were produced through biolistic bombardment transformation, showed a strong PCR band of ~2.2 kb (Fig. 3a and 3b). The ~2.2 kb PCR bands from these two vines were gel-purified, cloned, and sequenced. All clones showed identical sequences with 8 bps extra deletion at the 1176 target site and 3 bps extra deletion at the 1180 target site (Fig. 3d). Because all clones had identical sequences and shared the same editing features, we concluded that only one single event of double editing happened to these vines before the tissue sampled. In addition to the ~2.2 kb PCR band, a PCR band of ~12.5 kb was also amplified in the two plants. The presence of this 12.5 kb band suggests that these two vines most likely maintained a heterozygous status for the *VvMybA1* locus, with the *Gret1* present in one *VvMybA1* allele and removed in the other allele. We further observed the same ~12.5 kb and ~ 2.2 kb PCR bands from two additional leaf samples, which were collected about 3 weeks after the first leaf sample collection, and one root sample from each of the two vines (Fig. 3c). They all produced strong *Gret1*-free *VvMybA1* bands with comparable band intensities, further indicating that these two plants were likely derived from embryogenic cells with the *Gret1* edited out from one of their *MybA1* alleles. Becaues these two vines shared the exact editing profiles, we concluded that they were clones and derived from a single editing event.

To examine if the edited *VvMybA1* promoter in p4aNNRR2.1 or p4aNNRR3.1 is functional, we did RT-PCR assay of *VvMybA1* expression in leaves as well as shoots regenerated by *in vitro* propagation from the original P4aNNRR3.1 vine. For negative control, we used leaf tissues from another transgenic vine from the same experiment, CCNR2.3, which was tested positive for *Cas9* gene and hygromycin-resistant marker but with no detectable *Gret1* deletion in the *VvMybA1* promoter by genomic PCR. Because *VvMybA1* is primarily expressed in berries and stressed tissues but very low in leaf/shoot tissues, 45 cycles were used for RT-PCR reactions. p4aNNRR3.1 samples (both leaf and shoot) showed an unique band which was not present in CCNR2.3 samples (Fig. 3e). The band was an amplicon of *VvMybA1* between the PCR primers confirmed by cloning and sequencing of the unique gel band from multiple samples. This data suggests that the gene-edited *VvMybA1* in p4aNNRR3.1 vine (Allele b in Fig. 3d) indeed had the *MybA1* promoter function restored. Furthermore, we conducted a leaf color assay and observed that leaf samples from p4aNNRR3.1 showed apparent coloration changes, compared with the control (Fig. S4). This leaf color change indicated that p4aNNRR3.1 would likely produce berry with red color since leaf coloring and berry coloring are highly correlated [37].

### Characterization of editing events in *VvMybA1* locus using high-throughput sequencing

To obtain an overall assessment of double editing events, amplicon libraries were constructed and sequenced from the ~2.2 kb PCR bands amplified from those Cas9-positve vines with the primer pair 1450 and 1160 as described earlier. Many Cas9-positve vines didn’t produce the ~2.2 kb PCR bands. We selected 12 vines transformed by *Agrobacterium* and 11 vines (including p4aNNRR2.1 and p4aNNRR3.1) transformed by biolistic bombardment which showed some amplifications of the ~2.2 kb band (Fig. 3b) for amplicon library construction and high throughput sequencing. In addition, 5 plates of callus clusters from *Agrobacterium* and bombardment transformation were included in the analysis. High-throughput sequencing of the amplicon libraries revealed many double editing variants in these samples (Tables 1 and S3). By compiling top 50 most-frequently observed variants from each sample, 145 variants were detected. Among the 20 top-ranked variants across all samples, DE1 was the most observed double-editing variant (Tables 1 and S3). DE1 was the same Type 1 variant as observed in the callus sample 291 discussed earlier (Fig. 2b) and had perfect end joining between the two target sites of the 1176 and 1180. This perfect editing variant was detected in 21 of the 28 samples studied with an average indel percentage of about 15.2% (Table 1). Many editing variants belonged to Type 2 (Fig. 2b) with various sizes of deletions at the editing sites (Tables 1 and S3). For example, DE2 was the second most common variant with 11 bps extra deletion and an average percentage of about 12.4%, compared to the Type 1 or DE1 perfect editing variant (Tables 1 and S3), and was primarily detected in the bombardment-transformed callus and vines. This variant was the same double-edited *VvMybA1* allele in p4aNNRR2.1 and p4aNNRR3.1 based on cloning and Sanger sequencing discussed above. As expected, amplicon sequencing of p4aNNRR2.1 and p4aNNRR3.1 revealed that DE2 was the only double editing variant detected in these two vines. In contrast, many different types of double editing variants were found in the other vine samples. We detected some variants with extra deletion more than 50 bps, such as DE10. We also observed variants with various sizes of insertion, ranging from one or few bps to several dozens of bps. For example, DE18 had 32 bp insertion in addition to 27 bp deletion. However, this variant was observed in only about 0.44% of all mapped reads. Furthermore, we observed a large range of percentage variation for the same variants between callus and vine samples and even within the same type of tissue as indicated by the observed maximum and minimum indel percentages (Table 1). The amplicon sequencing also uncovered occurrences of some Type 3 variants in some callus and vine samples but with a percentage of 0.5% or lower. Among the 67 *Agrobacterium*- and 24 biolistic bombardment-transformed vines with the presence of the Cas9 transgene, we found only two vines having the *Gret1* edited out. These vines were most likely derived from the same edited embryogenic cells because they shared the same editing profiles. There are certainly many factors contributing to such low double editing efficiency and target sites are certainly important ones. We evaluated six Cas9 target sites for the *MybA1*–5’LTR junction and three target sites for the 3’LTR-*MybA1* junction for their editing efficiencies by *in vitro* CRISPR/Cas9 ribonucleoprotein (RNP) digestion (Fig. S1 and Table S2). Based on the *in vitro* RNP digestion results and the potential off-target matches of sgRNAs (Table S2), we decided to focus on the target sites 1176 and 1180 in this study. To provide an assessment of editing efficiencies of the individual target sites 1176 and 1180, we constructed amplicon libraries for analyzing individual target sites from 10 transgenic callus plate samples and 26 transgenic vine samples transformed by *Agrobacterium* and 22 from biolistic bombardment (Tables 2 and S5). These 48 transgenic vines included the 23 vines showing potential double editing described earlier. The top 4 variants from each of the 10 transgenic callus and the 48 vine samples were analyzed as representative variants and their average indel percentages or editing efficiencies were determined for the two target sites 1176 and 1180 in both callus and vine samples (Table 2).

**Table 1:** Indel percentages of top 20 most observed T1-T2 variants resulted from simultaneous editing of both target sites 1176 and 1180 in the transgenic callus and plants

Variant description^a^					*Agrobacterium*-transformed callus (2) and plants (12)			Biolistically-transformed callus (3) and plants (11)			
Sequence ID	Variant type	No. bps deletion at the 1176 target	No. bps deletion at the 1180 target	No. bps insertion at the junction	Mean^b^	Max^c^	Min	Mean	Max	Min	Overall mean
DE1	T1	0	0		21.71	40.14	8.49	8.67	**50.74**	0.00	15.19
DE2	T2	8	3		0.49	1.39	0.00	24.22	**86.64**	0.00	12.36
DE3	T2	4	35		0.68	2.95	0.00	5.88	**82.16**	0.00	3.28
DE4	T2	1	0		3.66	11.01	0.01	1.88	12.45	0.00	2.77
DE5	T2	23	0		3.79	14.47	0.02	0.64	8.97	0.00	2.22
DE6	T2	2	0		1.60	8.00	0.00	1.89	13.43	0.00	1.75
DE7	T2	0	1		2.44	8.06	0.01	0.58	7.29	0.00	1.51
DE8	T2	2	2		1.64	3.60	0.00	0.42	5.91	0.00	1.03
DE9	T2	4	0		0.86	7.07	0.00	1.04	14.58	0.00	0.95
DE10	T2	64	3		1.87	26.10	0.00	0.00	0.00	0.00	0.93
DE11	T2	2	1		0.52	2.28	0.00	1.30	13.77	0.00	0.91
DE12	T2	48	0		1.69	23.58	0.00	0.00	0.00	0.00	0.85
DE13	T2	2	16		1.16	5.86	0.00	0.00	0.00	0.00	0.58
DE14	T2	23	9		0.02	0.22	0.00	1.05	7.84	0.00	0.54
DE15	T2	1	1		0.01	0.12	0.00	1.00	12.84	0.00	0.51
DE16	T2	3	46		0.99	12.84	0.00	0.00	0.00	0.00	0.49
DE17	T2	0	6		0.30	2.50	0.00	0.68	9.54	0.00	0.49
DE18	T2	27	1	33	0.00	0.00	0.00	0.89	12.44	0.00	0.44
DE19	T2	5	0		0.84	5.93	0.00	0.00	0.01	0.00	0.42
DE20	T2	4	27		0.78	5.07	0.00	0.00	0.00	0.00	0.39

a These variants were selected on the basis of their overall mean indel percentages within the editing window assayed across all 28 samples; see Figure 2 for the definition of editing variant types T1-T2

b The indel percentage for an individual sample was calcuated with the reads of a variant divided by its total mapped reads; the mean indel percentage was calculated as an average of all samples in the group of concern

c Maximum indel percentages of 50% or more highlighted in bold and they were all from bombardment-transformed callus or vines.

**Table 2:** Representative variants and their indel percentages observed at the individual Cas9 target sites 1176 and 1180 in the transgenic callus and vine

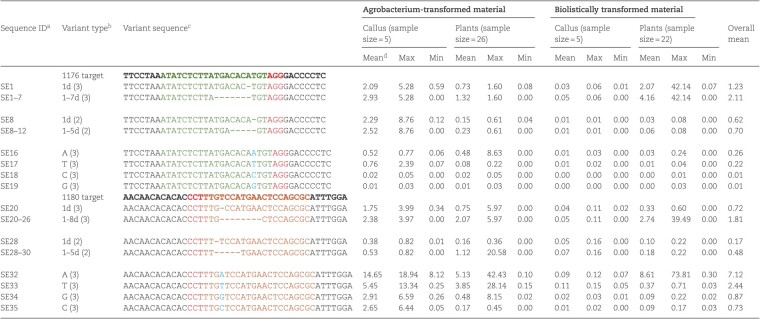

a Sequence ID is the same as in Table S4

b Variant types were described on the basis of the changes of base pairs using the relevant PAM site as a reference. For example, “1d (3)“satnds for 1 bp deletion and “1–7d (3)“for the sum of 1–7 bp deletion at 3 bp upstream of the PAM site; “A (3)“stands for insertion of “A” at the postion of 3 bps upstream of the PAM site

c The 3 bps of the PAM sites highlighted in red, the 20 bps before the 1176 PAM site in green and 1180 PAM site in brown, insertion bps in blue, and deleted bps represented by “-”

d The indel percentage for an individual sample was calcuated with the reads of a variant divided by its total mapped reads; the mean indel percentage was calculated as an average of all samples in the group of concern.

The most common variants across all samples were small indels, such as SE32 (A insertion), SE33 (T insertion), SE1 (1 bp deletion) or 1–8 bp deletion at 3 bps upstream of the PAM sequences for both target sites (Tables 2 and S4). It was noted that most editing took place at the 3 bps upstream of the PAM sequences, as theoretically predicted, but many editing events also occurred at the 2 bps upstream (Tables 2 and S4). In contrast, editing at the 1 or 4 bps upstream of PAM sequences was much rare (Table S4). Furthermore, among the editing events with single bp insertions, “A” and “T” were more frequently observed than “G” and “C” consistently across both target sites (Tables 2 and S4). Overall, the average indel percentages differed significantly between the 1176 and 1180 target sites in both callus and plant samples and also between *Agrobacterium* transformed and bombardment transformed samples. The mean indel percentage for the five *Agrobacterium*-transformed callus samples is close to 50% for 1180 site, more than five times of the mean editing efficiencies for the 1176 site (49.46% vs 8.81%). The editing efficiencies for both 1176 and 1180 target sites were very low in bombardment-transformed callus samples, but the difference between the two target sites is still about 5 times (0.10% vs 0.56%). The difference in averaged editing efficiencies for 1176 and 1180 sites was still large for transgenic plants, 19.85% for 1180 and 2.80% for 1176 in 26 *Agrobacterium-*transformed vines, 16.19% for 1180 and 5.05% for 1176 in 22 bombardment-transformed vines (Table S4). Interestingly, there was a large difference of indel percentages between the two transformation methods in transgenic callus, but not so much in transgenic vines (Table 2). Presumably this was because a large portion of bombardment callus contained the hygromycin marker but didn’t carry the gene editing components, as supported by the findings that more than 75% bombardment-transformed vines were tested *Cas9* negative.

Based on the editing percentages of individual target sites in a transgenic vine, it was possible to determine if the vine was regenerated from an edited embryogenic cell. If editing took place in one of the alleles in an embryogenic cell from which a vine was regenerated, we would expect that the editing percentage for a given variant in the vine should be 50% or more. This is because a constitutive promoter was used for driving the editing components in MG1 and additional editing could take place in the other allele in somatic cells. Among the 26 *Agrobacterium*-transformed plants studied, we did not find any vines with a single variant having a percentage of more than 50% for either target site (Table 2), suggesting all these vines were likely regenerated from non-edited embryogenic cells. Among the 22 bombardment-transformed plants, p4aNNRR2.1 and p4aNNRR3.1 both had a single dominant variant of “A” insertion with the percentage in the 1180 target site of over 73% (Table 2). When all reads were considered, the “A” insertion in the 1180 target site was found in more than 95% of mapped reads across four replicates of the two vines (Table S5). Since these two vines had one *VvMybA1* allele with the *Gret1* removed based on long amplicon genomic PCR and Sanger sequencing (Fig. 3), this high editing percentage for the 1180 site must come from a single editing event of the second *VvMybA1* allele. Based on these evidence, both p4aNNRR2.1 and p4aNNRR3.1 vines had one *VvMybA1* allele with the *Gret1* removed through double editing of both 1176 and 1180 target sites and the other *VvMybA1* allele with an insertion of “A” through single editing of the 1180 target site and the *Gret1* not removed. Both *VvMybA1* alleles were likely edited at the embryogenic cell stage.

## Discussion

### *VvMybA1* editing and creation of new functional recombinants of *VvMybA* alleles

Due to the presence of three *VvMybA*-like genes and a *Gret1* transposon in the *VvMybA1*, the *VvMybA* berry color locus in grape cultivars often shows spontaneous structural variation [5, 15]. *Gret1* is bordered with two similar LTRs between which an intra-LTR recombinant could result in removing the *Gret1* and leaving a functional *VvMybA1* with one copy of LTR. Several such natural recombinants were reported and found responsible for the changes in berry color [5, 8, 38–40]. In this study, we introduced changes into *VvMybA1* locus via CRISPR/Cas9-based editing and observed extensive structural changes in the locus, including the removal of whole *Gret1* transposon from *VvMybA1* by simultaneously targeting two *Gret1* flanking sites as well as the formation of *VvMybA3*/*VvMybA1* and *VvMybA2*/*VvMybA1* hybrid structures (Fig. 2, Tables 1 and S3). Presumably, the CRISPR-Cas9 editing machinery created breaks in the *Gret1* transposon/*VvMybA1* junction sequences, then the breaks were either repaired by NHEJ or by homology-based repairing involving highly homologous *VvMybA1*, *VvMybA2* and *VvMybA3* (Fig. 2). Furthermore, the gRNA for the target site 1180 in *VvMybA1* could potentially target *VvMybA3* and *VvMybA2* genes because of the high similarities of the target site sequences among these three genes with only one or two mismatches (Fig. 2b). Potentially, a DNA break could be created in *VvMybA3* or *VvMybA2* besides a break in *VvMybA1*, and a homology-based repairing mechanism could lead to the formation of various hybrid structures of *MybA* genes. Such editing-induced homologous recombination is expected to occur at a much higher frequency than that in nature and therefore could offer unique opportunities for recovering multiple functional *MybA*-like alleles, similar to how the functional *VvMybA1* locus was formed in the color bud sport “Benitaka” from “Italia” [11]. Similar hybrid genes between *TAS4a* and *TAS4b* created by gene editing and homologous recombination have recently been reported in grape [20]. While we demonstrated in this study the possibility of knocking out the *Gret1* from a *VvMybA1* allele through gene editing for possibly restoring the *VvMybA1* function, opportunities for enhancing *VvMybA1* expression through editing, thus anthocyanin formation and accumulation, are also present. For example, repeats of a 408-bp fragment in a *VvMybA1* promoter were found to enhance *VvMybA1* expression in teinturier grapes [41] and we could knock in such repeats in the promoter region of a functional *VvMybA1* allele for enhancing its expression.

### Successful removal of the *Gret1* transposon from and functional restoration of a *VvMybA1* allele

To remove a large DNA fragment through gene editing, the two target sites need to be cut at the same time and the two breaks, after the DNA fragment is removed, need to be repaired by joining the non-homologous ends together [27]. In this study we produced two vines p4aNNRR2.1 and p4aNNRR3.1 with a nucleotide “A” inserted in one of their *VvMybA1* alleles through single site editing at the 1180 target site and the *Gret1* transposon removed from the other *VvMybA1* allele through double editing of both 1176 and 1180 target sites. These two vines were most likely clones and derived from the same edited cell because they had the same editing profiles and the chance to obtain the same unique editing features through independent editing events was extremely unlikely, especially considering that duplicate clones are often regenerated during transformation and tissue culture processes. Based on its editing efficiencies, we believe that the double editing event in p4aNNRR2.1/p4aNNRR3.1 most likely occurred at the single embryogenic cell or very early embryogenic callus stage. While we cannot demonstrate without any doubt that p4aNNRR2.1/p4aNNRR3.1 contained no chimeric cells, we have compelling evidence to suggest that p4aNNRR2.1/p4aNNRR3.1 was most likely heterozygous regarding the *Gret1* editing. If p4aNNRR2.1/p4aNNRR3.1 was chimeric, we expect that *Gret1* editing would most likely continue in the cells whose *VvMybA1* had not been edited. Therefore, different types of edited *VvMybA1* alleles would be detected through high throughput sequencing and their frequencies would vary from tissue to tissue and with different developmental stages. On the other hand, if p4aNNRR2.1/p4aNNRR3.1 was heterozygous, we expect that two main types of alleles (with or without the *Gret1*transposon) would be observed, although in theory the Gret1-containing allele would continue to be subjected to further editing. As presented in Figure 3c and 3d, we found only two types of *VvMybA1* alleles in p4aNNRR2.1/p4aNNRR3.1 through amplicon sequencing as well as cloning and sequencing of PCR bands from different types of tissues (leaves and roots) sampled at different time points.

Although *VvMybA1* is primarily expressed in berry skin, we were able to detect *VvMybA1* expression in the p4aNNRR3.1 tissue samples by RT-PCR with *MybA1*-specific primers (Fig. 3e). Funtional restoration of the edited *VvMybA1* was further demonstrated in the leaf coloring assay. Precise removal of more than a 10-kb DNA fragment from the *VvMybA1* locus and restoration of its function in this study broaden the possibilities of using gene editing technologies to modify various trait genes in grapes and other plants.

### Enhancement of double editing efficiencies

We were not surprised that double editing events for removal of the *Gret1* transposon from *VvMybA1* in transgenic callus and vines were not common. *Gret1* is more than 10 kb long and removal of such a large piece of DNA fragment is certainly challenging, given that editing efficiencies are generally lower as the intended deletions are larger [30]. Significant enhancement of double editing outcomes would likely require better optimization/improvement in the CRISPR-Cas9 editing machinery through the use of efficient target sites, strong promoters for *Cas9* and sgRNA expression [42], *Cas9* with intron for improving editing efficiencies [43], and enhanced sgRNA (esgRNA) design [44]. Among all these factors, target sites appeared to be most critical. In this study, we had limited options for choosing target sites for removal of the *Gret1* transposon from *VvMybA1* because the target sequence window between *Gret1* and the *VvMybA1* promoter was rather narrow, especially when we tried to minimize off-target editing and potential negative impact from the deletion of some critical base pairs from the *VvMybA1* promoter sequence (Table S2). While *in vitro* RNP digestion of the sequence fragments containing *MybA1*- *Gret1* junctions suggested similar cutting efficiencies for the two target sites (1176 and 1180) (Fig. S1), the average editing efficiencies for the target site 1180 were consistently higher than that for 1176 in transgenic callus and vines and the differences could be as high as 5 folds or more (Table 1). The higher editing efficiency at the 1180 site was also observed when a *AtU3* promoter was used to drive tandem tRNA-esgRNA1176 and tRNA-esgRNA1180 units (data not shown). These results highlighted the critical importance of choosing effective target sites for gene editing, especially for simultaneous editing of two target sites. In addition to construct optimization, the use of highly transformable plant materials such as protoplasts [45], embryos [46], and meristematic bulks [47, 48] and efficient transformation methods such as the use of ribonucleoproteins [45] for gene editing are also crucial.

An alternative approach to regain berry color is to restore *VvMybA2* function in “Chardonnay” or other white grape cultivars. *VvMybA2* appears to have stronger activities than *VvMybA1* [7] and restoring *VvMybA2* function might create a cultivar with deeper coloration than restoration of the *VvMybA1* locus. *VvMybA2* is expressed in white berry skin, but its coding region was mutated and truncated. With the promise of prime-editing and other new editing technologies [49, 50], it is possible to repair the mutated *VvMybA2* coding region. Moreover, the use of a CRISPR-Cas9 editing system coupled with a homologous repair template could also be potentially utilized to replace a mutated *VvMybA2* with a functional *VvMybA2* [51, 52]. Most gene editing has so far been achieved through expression of Cas9 and other editing components in transgenic plants. Because of various concerns associated with genetically modified organisms, it is highly desirable to edit a gene without leaving any transgenic footprints. For inbreeding and highly homozygous crop species, transgenic components associated with gene editing can be segregated out through selfing or crossing. However, for outcrossing and highly heterozygous species such as grapevine, selfing or crossing will unavoidably change the whole genome composition (i.e. genetic identity) of the genotype involved in the editing. Non-transgenic editing approaches have actively been pursued. Recently, a transient editing approach has been successfully applied to protoplast in grapevine [45], offering a promising future to edit a gene non-transgenically in grapevine.

## Materials and methods

### Vector construction

To remove the *Gret1* transposon from a *VvMybA1* locus, which is clustered with *VvMybA2* and *VvMybA3* (Fig. 1a), a vector with dual-sgRNA/Cas9 design was constructed as follows (Fig. 1b and 1c). The p201H vector (Addgene #59176) containing a Cas9 driven by CaMV 35S promoter was obtained from Addgene [31]. To clone two sgRNA cassettes into the p201H binary vector, SwaI_MtU6F and NS1_ScaffoldR were used to amplify the MtU6:sgRNA1 cassette while UNS1_MtU6F and SpeI_ScaffoldR were used to amplify the MtU6:sgRNA2 cassette (Table S1). The vector components were assembled following the Gibson assembly approach using SwaI/SpeI digested p201H, sgRNA1176 cassette and sgRNA1180 cassette resulting in the final *VvMybA1* gene editing construct, p201H-MtU6:1176-MtU6:1180 (named as MG1) (Fig. 1c). sgRNA1176 and sgRNA1180 were chosen based on the *in vitro* Cas9 target site analysis (Table S2 and Fig. S1).

### Grape transformation and plant regeneration and propagation

Embryogenic callus was induced from inflorescences of *V. vinifera* cv. Chardonnay as previously reported [32]. Inflorescences were taken in May from field-grown Chardonnay which has been maintained for many years in the USDA-ARS table grape breeding program in the San Joaquin Valley Agricultural Sciences Center, California. The embryogenic callus was maintained on a callus induction medium (CIM) containing 4.49 g/L C287 (Chee & Pool C2D Vitis medium), 1 ml/L Murashige & Skoog modified vitamin mix (M557), 0.75 g/L casein, 30 g/L sucrose, 0.2 mg/L 6-BAP, and 1.1 mg/L 2,4-D, and 7.5 g/L agar (A175) adjusted to pH 5.8 before autoclaving. Unless specified, callus was incubated in dark at room temperature. The *Agrobacterium tumefaciens* EHA105 strain harboring the binary construct (MG1) was used to transform grape embryogenic callus [33]. The Agro-infected callus was incubated on a filter paper in CIM plates supplemented with 100 μM acetosyringone for ~3 days and then washed 5–7 times with sterile liquid CIM medium, followed by 1–2 times of final wash with CIM medium supplemented with 200 μg/ml Timentin to remove the Agrobacterium. Subsequently, the washed callus was incubated on CIM plates supplemented with 100 μg/ml Timentin for a week and then transferred to callus selection medium (CIM supplemented with 100 μg/ml Timentin and 10 μg/ml hygromycin B). The callus was transferred to fresh selection medium every 3–4 weeks until antibiotics-resistant sectors appear. Three to six transfers were needed until hygromycin-resistance callus sectors became prominent. Once robust callus was established, some calli were transferred to embryo-induction medium with 5–10 μg/ml hygromycin B (CIM-NH plates: 4.49 g/L C287; 1 ml/L M557 vitamin solution; 0.75 g/L Casein hydrolysate; 60 g/L sucrose; 2.5 g/L active charcoal; pH 5.8; 10 g/L Agar (A175)). The calli were transferred to fresh CIM-NH plates every 3–4 weeks until somatic embryos were induced. Once embryos were induced, they were transferred to C2D4B plates for embryo germination under light (16 hours of light followed by 8 hours of darkness) (4.49 g/L C287; 1 ml/L M557 solution; 30 g/L sucrose; 0.9 mg/L 6-BAP; pH 5.8; 7 g/L agar (A175)). After 3–4 weeks, the green germinated embryos were transferred to rooting medium in a Magenta box (4.43 g/L M519; 30 g/L sucrose; 0.09 mg/L NAA; pH 5.8; 7 g/L agar (A111)). After one to two months of culture in a Magenta box under light, plants were transferred to soil in 4-inch pots covered with plastic bags to maintain humidity. Well established plants were repotted into one gallon pot and maintained in a greenhouse. All tissue culture reagents were purchased from PhytoTechnology Laboratories (PhytoTech Labs, Inc., Lenexa, KS, USA).

To deliver the binary vector (MG1) into embryogenic callus by biolistic transformation method, embryogenic calli 5–7 days on fresh CIM plates were subjected to an osmotic treatment for four hours before bombardment on osmotic medium (CIM plus 0.2 M mannitol, and 0.2 M sorbitol). For particle bombardment, 150–200 yellowish and compact calli were arranged in a 2.5 cm diameter circle at the center of a plate to maximize the DNA delivery from the particle delivery process. Biolistic transformation of embryogenic calli was performed based on the protocol by Vidal [34] with minor modifications as described below. Sixty milligrams of 0.6 μm gold particles were washed twice with 200-proof ethanol and rinsed once with sterile water. Washed gold particles were resuspended in 1 ml of 50% sterile glycerol solution and aliquoted 50 μl into each microfuge tubes and stored at −20°C. For DNA coating, 200 ng of plasmid DNA were added to the tube containing 50 μl aliquot of gold particles first, then sequentially mixed with 20 μl glycogen (20 mg/ml), 50 μl 2.5 M CaCl₂, and 20 μl of 0.5 M spermidine. Subsequently, the mixture was put on ice-bath for 30 min, the gold particles were collected by gentle centrifugation and washed thoroughly with 200-proof ethanol. The washed DNA-coated gold particles were resuspended in 120 μl 200-proof ethanol and used for 6 shots. Following bombardment, the calli were kept on the original plate overnight and then transferred to fresh CIM media without selection. After one week, the calli were transferred to selection media containing10–20 mg/L hygromycin. Once hygromycin-resistant calli were established, embryo induction and plant regeneration were conducted as described above.

To multiply specific transgenic vines *in vitro*, internode cuttings with at least one axillary/lateral bud were incubated on a propagation medium containing MS with vitamins, 4.4 μM benzyl adenine (BA), 1 μM thidiazuron, 3% sucrose, and 0.7% agar (pH 5.6) initially for 3 weeks to allow recovery and shoot growth from the buds and then transferred to fresh propagation medium for further proliferation. The proliferating green shoots were again trimmed, and the cuttings placed on propagation medium containing 4 μM BA, 3% sucrose, and 0.7% agar (pH 5.6) to further multiply the shoots. The resulting healthy green shoots were transferred to shoot elongation medium containing MS with vitamins, 2 μM BA, 3% sucrose, and 0.7% agar (pH 5.8) for 3–4 weeks followed by rooting on medium containing MS with vitamins, 4.92 μM Indole-3-butyric acid, 3% sucrose, and 0.7% agar (pH 5.8). Multiple green shoots were used for RNA extraction and RT-PCR.

### LongAmp genomic PCR

We used LongAmp® Taq PCR kit (NEB, E5200S) to amplify the *MybA1* locus. Ten to fifty nanograms of grape genomic DNA extracted with Qiagen DNeasy Plant kit was used in a 25-μl reaction volume, 5 μl 5x LongAmp Taq reaction buffer, 0.75 μl 10 mM dNTPs, 1 μl 10 μM forward primer, 1 μl 10 μM reverse primer and 1 μl LongAmp Taq DNA polymerase. The PCR cycling condition included an initial denaturation at 94°C for 30 seconds, followed by 30 cycles at 94°C for 30 seconds, 51°C for 30 seconds, and 65°C for 11 minutes, and ended with 65°C for 10 minutes with primer pairs 1450/1160 or 1153/1160 (Fig. 1). Primer 1160 is specific to *VvMybA1* and located in its terminator region while primer 1450 can bind to *VvMybA1* with perfect match but also likely bind to *MybA3* with 1 bp mismatch. Primer 1153 can bind to all three *VvMybA* promoters with perfect match for the last 24 bp (Fig. 1 and Table S1). PCR amplification from a *Gret1*-containing *MybA1* is expected to be about 12 kb, while a gene-edited *MybA1* without *Gret1* is expected to be ~1.8 kb (1153/1160, Fig. 2) or ~ 2.2 kb (1450/1160, Fig. 3). The primer set of 1153/1160 can also detect editing variants involving *VvMybA2* and *VvMybA3*.

### Amplicon library construction

Two types of genomic amplicon libraries were constructed in this study. The first one was based on the 1^st^ round PCR products directly amplified from genomic DNA samples of transgenic callus or plants. For the 1^st^ round PCR, the primer pair 1154/1247 was used to amplify the *MybA1*–*5’LTR* junction sequence covering the 5′ target site 1176, and the primer pair1158/1246 was used to amplify the *3’LTR*-*MybA1* junction sequence covering the 3′ target site 1180 (Fig. 1a). These primers all had adaptors for annealing to second round bar-coded primers. 10–50 ng genomic DNA was used in a 15-μl reaction with Go-Taq polymerase (Promega) for 30 cycles. The 1^st^ round PCR products were purified with 1.2X AMPure XP beads (Beckman Coulter) and eluted in 15 μl water. 1–2 μl of purified 1^st^ PCR product was used for the 2^nd^ round PCR reaction with Phusion DNA polymerase (NEB) in a 20-μl reaction (8 cycles) with barcoded primers. The 2^nd^ PCR products were purified with 1X AMPure beads and eluted in 15 μl water. Library concentration was measured by NanoDrop One (Thermo Scientific). Individual libraries were pooled together based on their concentrations and intended coverage and sequenced using a Hi-Seq 2x150 bp sequencing platform by Genewiz or Biotechnology Resources Center at Cornell University.

The second type of genomic amplicon libraries constructed was from a ~ 2.2-kb DNA band from long amplicon genomic PCR with the primer set 1450/1160. The targeted DNA band, presumably resulted from double-edited *VvMybA1* with the *Gret1* removed, was gel-purified and used as the substrate for the 1^st^ round PCR for amplicon library construction with the primer set 1154/1246, which covered the *Gret1*/*MybA1* junctions including the two Cas9 target sites (Fig. 1a). The rest of steps were similar to those for regular genomic amplicon library construction as previously described.

### Amplicon sequence data analyses

Amplicon sequence data were analyzed using Cas-Analyzer (http://www.rgenome.net/cas-analyzer/) [35]. For single target site analysis, the genomic sequence between primer pairs was used as the wild type (WT) sequence. The reference sequence for the 5′ target site is shown as follows: ggacgttaaaaaatggttgcacgtggttgccttcaggatcacaccagtttatacatttggaccacaaaatagagattgttcatcaaggatactagtcagcaattaattcctaaatatctcttatg**acaca***tgt**agggacccctccctctgggaaacacgtggcacgcacctcacagtgacacgcagcacgtgttatcagccggaccatcatcatccggattcccttaaggatacgcatgatgatggttctcc* with the target duplication sequence (TSD) in bold, the 5’LTR sequence in italic, and the 1176 target site underlined. The reference sequence for the 3′ target site is *tcctctctctaaccatggctaacaaaaccatcggaggatgcgtccagacaccctgtccggatgccttcttgcaggaatgacgactggatcaaaaacctttatgagttgagatcacgcgtccatccatctggttactacgtggaccgccaaagacgcgaggtaacaacaac***acaca**ccctttgtccatgaactccagcgcatttggaagccagtaatgcaccataagaaacgtgtcgaataaaccaattaggggtctggtgtccgagtcatgagatag with TSD in bold, 3’LTR sequence in italic, and the 1180 target site underlined. Paired-end reads were analyzed using “45 bp” as the comparison range, with “1” as the minimum frequency and the default “5 bp” for the wildtype marker. Total reads, mapped reads, reads with insertion or deletion and total indel frequencies as well as detailed mutations were generated by Cas-Analyzer. Detailed mutation variants and read numbers were exported from Cas-Analyzer for analyzing specific variants and frequencies. Amplicon from non-transgenic callus was also analyzed as the background control. The mean numbers of mapped reads for callus and vines samples were 1,369,801 and 298,190, respectively.

For the analysis of the amplicon data derived from Long Amp PCR, the paired end reads were merged using Qiagen CLC genomic workbench. The merged reads were sorted, based on sequencing identity and abundancy, and analyzed manually. The amplicon data were also analyzed by Cas-Analyzer for variant types and frequencies of mutational changes at the *Gret1*/*MybA1* junctionsusing the sequence of an ideal double editing event as the reference: ggacgttaaaaaatggttgcacgtggttgccttcaggatcacaccagtttatacatttggaccacaaaatagagattgttcatcaaggatactagtcagcaattaattcctaaatatctcttatg**acaca**tccatgaactccagcgcatttggaagccagtaatgcaccataagaaacgtgtcgaataaaccaattaggggtctggtgtccgagtcatgagatag with the mock-up target sequence underlined. This reference sequence was predicted from an edited *VvMybA1* with the *Gret1* removed by simultaneously creating and rejoining breaks at 3 bps upstream from the PAM sites of both sgRNA1176 and sgRNA1180 targets(Fig. 1b). After multiple trials with different comparison ranges and mock-up target sequences, we found that results with 85 bp as the comparison range and the mock-up target sequence(underlined in the reference) were most close to the manualsorting results and the mapped reads ranged from 89% to 99.9% of total reads across different samples. The mean numbers of mapped reads for callus and vines samples were 254,724 and 116,892, respectively. Results from Cas-Analyzer with these parameters were summarized for top-ranking editing variants in different transgenic materials.

### RNA extraction, RT-PCR, PCR band cloning, colony PCR and sequencing

Leaves from transgenic vines in the margenta boxes or shoots on propagation plates were collected and grounded in liquid nitrogen. Plant Spectrum total RNA kit from Sigma was used to extract total RNA. RNA concentration was measured by Nanodrop One. Trace of genomic DNA in the total RNA samples was removed by DNA-free DNA removal kit (AM1906, ThermoFisher Scientific). For each sample, ~ 500 ng DNase-treated total RNA was used for reverse transcription (RT) in 20 μl volume with d(T)23 VN primer using the Postscript II first strand cDNA synthesis kit (E6560, New England Biolabs). For each RT-PCR reaction, 0.5 μl RT reaction was used in a 20 μl-reation with GoTaq DNA polymerase (Promega). Because of high sequence similarities among *VvMybA1*, *VvMybA2* and *VvMybA3* genes, we designed a pair of primers (1470 and 1563) which can specifically amplify *VvMybA1*, but not *VvMybA2* or *VvMybA3*. Specifically, the last base “**C**” in primer 1470 (CTATTGGCATAGTCACCACTa**C)** is unique to *VvMybA1* and the last but one base “a” does not match with any of the three *VvMybA* genes. It is known that a primer with only one base mismatch could still get a target sequence amplified, but not likely with two mismatches. Therefore 1470 primer would amplify a target sequence only in *VvMybA1*. Similarly, the last base “**G**” in primer 1563 (GGCTT**C**CTGGAAGTACa**G)** is *VvMybA1*-specific, and the last but one base “a” does not match with *MybA1* nor *MybA2* while the entire primer 1563 is missing in *VvMybA3*. Primers 1560 (TTCTCGTTGAGGGCTATTC) and 1561 (CACAGACTTCATCGGTGAC) were used for RT-PCR amplification of a house keeping gene, glyceraldehyde-3-phosphate dehydrogenase (GAPDH). The PCR cycling conditions included an initial denaturation at 95°C for 3 minutes, followed by 45 cycles at 94°C for 30 seconds, 52°C for 30 seconds, and 72°C for 45 seconds, and ended at 72°C for 5 minutes. Four to six replicates were carried for each specific reaction. Two representative samples were presented. To confirm RT-PCR band identities, specific PCR bands were exercised and purified from agarose gel using the Monarch DNA Gel Extraction Kit (T1020, New England Biolabs). The purified PCR band was cloned into pJET1.2 vector using the CloneJET PCR cloning Kit (K1231, ThermoFisher Scientific). pJETF and pJETR were used for colony PCRs to amplify the inserts. Colony PCRs were examined on gel. PCR samples with appropriate size were purified by 1.2x Ampure beads and sequenced with the pJETR primer. The PCR sequencing results were analyzed by Sequencher.

## Acknowledgement

We would like to thank Dongyan Zhao of Cornell University for assistance with data analysis, Della Cobb of Cornell University, Amy Szewc-McFadden and Xia Xu of USDA-ARS Grape Genetic Research Unit for generating and maintaining embryogenic callus, Craig A. Ledbetter of USDA-ARS San Joaquin Valley Agricultural Sciences Center for providing Chardonnay inflorescences for embryogenic callus induction. John Ke and Wegi Wuddineh were participants of the ORISE-ORAU Education and Training Program. This project was partially supported by the American Vineyard Foundation.

## Author Contribution

The project was conceived and managed by G.-Y.Z. and designed by Y.Y., G.-Q. S. and G.-Y. Z.; Y.Y., X.H. and G.-Q.S. conducted *Agrobacterium* transformation, tissue culture and plant regeneration; J.K. did bombardment transformation and tissue culture and Y.Y. regenerated plants; W.W. did tissue culture and plant propagation; Y.Y. designed the constructs, performed PCRs, library construction and molecular analysis; Y.Y. and G-Y.Z. wrote the manuscript; and all contributed to the data interpretation and manuscript preparation. This project was partially supported by the American Vineyard Foundation.

## Data Availability

All amplicon data are available upon request.

## Conflicts of Interest

The authors declare that no competing interests exist.

## Supplementary data

Supplementary data is available at *Horticulture Research* online.

## Supplementary Material

supp_data_uhac201
