# Supplementary material for: Removal of a 10-kb Gret1 transposon from VvMybA1 of Vitis vinifera cv. Chardonnay
Source: Hortic Res. 2022 Sep 6;9:uhac201. doi: 10.1093/hr/uhac201 (PMC9669667; doi:10.1093/hr/uhac201)
Supplement: supp_data_uhac201 [file supp_data_uhac201.zip › Supplementary Figures 8.20.2022 .pptx]

## Slide 1
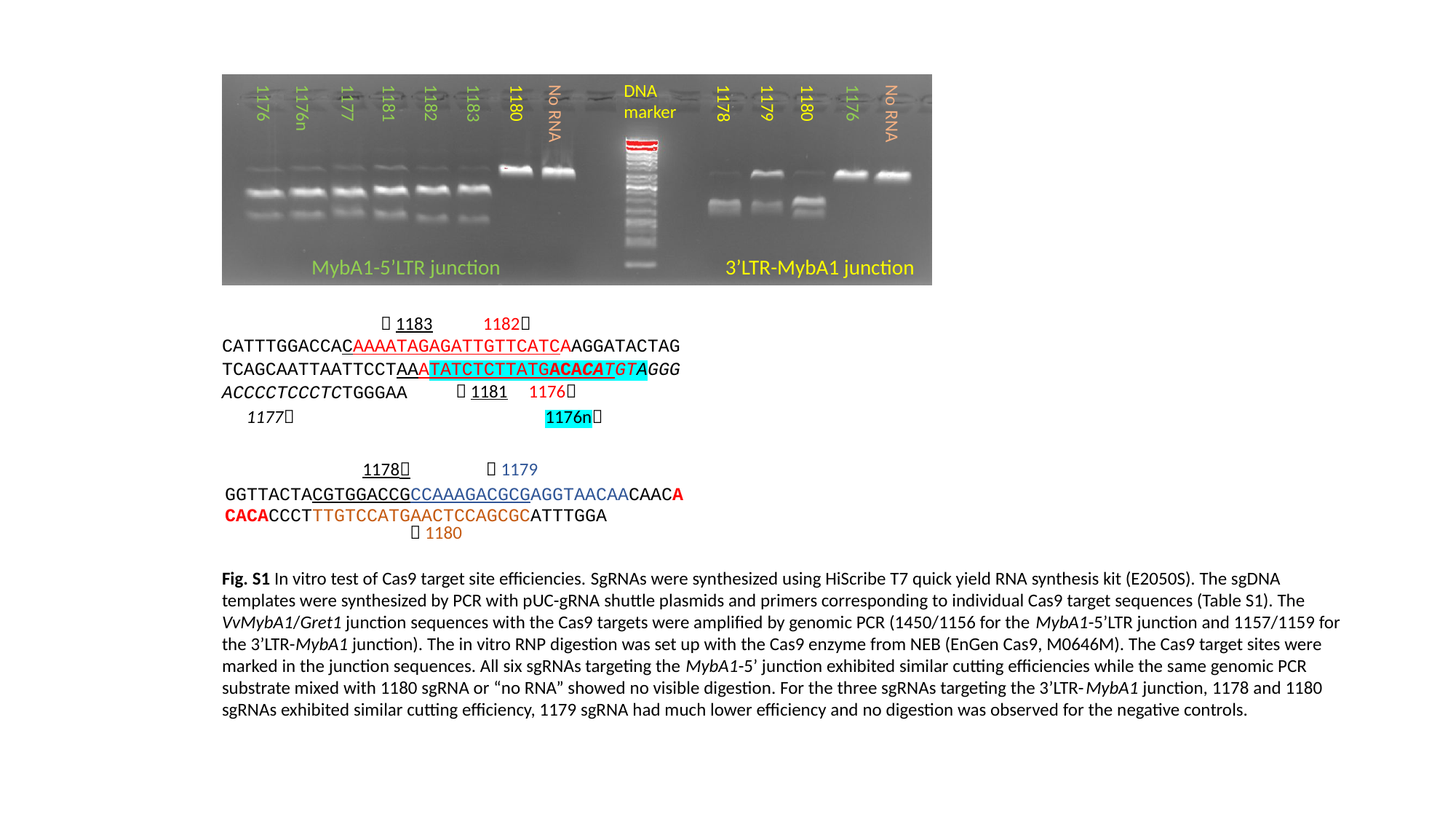

DNA marker
1176
1177
1181
1182
1183
1180
1178
1179
1180
1176
1176n
No RNA
No RNA
MybA1-5’LTR junction
3’LTR-MybA1 junction
  1183 1182
catttggaccacaaaatagagattgttcatcaaggatactagtcagcaattaattcctaaatatctcttatgacacatgtagggacccctccctctGGGAA
  1181 1176
 1177
 1176n
 1178  1179
GGTTACTACGTGGACCGCCAAAGACGCGAGGTAACAACAACACACACCCTTTGTCCATGAACTCCAGCGCATTTGGA
  1180
Fig. S1 In vitro test of Cas9 target site efficiencies. SgRNAs were synthesized using HiScribe T7 quick yield RNA synthesis kit (E2050S). The sgDNA templates were synthesized by PCR with pUC-gRNA shuttle plasmids and primers corresponding to individual Cas9 target sequences (Table S1). The VvMybA1/Gret1 junction sequences with the Cas9 targets were amplified by genomic PCR (1450/1156 for the MybA1-5’LTR junction and 1157/1159 for the 3’LTR-MybA1 junction). The in vitro RNP digestion was set up with the Cas9 enzyme from NEB (EnGen Cas9, M0646M). The Cas9 target sites were marked in the junction sequences. All six sgRNAs targeting the MybA1-5’ junction exhibited similar cutting efficiencies while the same genomic PCR substrate mixed with 1180 sgRNA or “no RNA” showed no visible digestion. For the three sgRNAs targeting the 3’LTR-MybA1 junction, 1178 and 1180 sgRNAs exhibited similar cutting efficiency, 1179 sgRNA had much lower efficiency and no digestion was observed for the negative controls.

## Slide 2
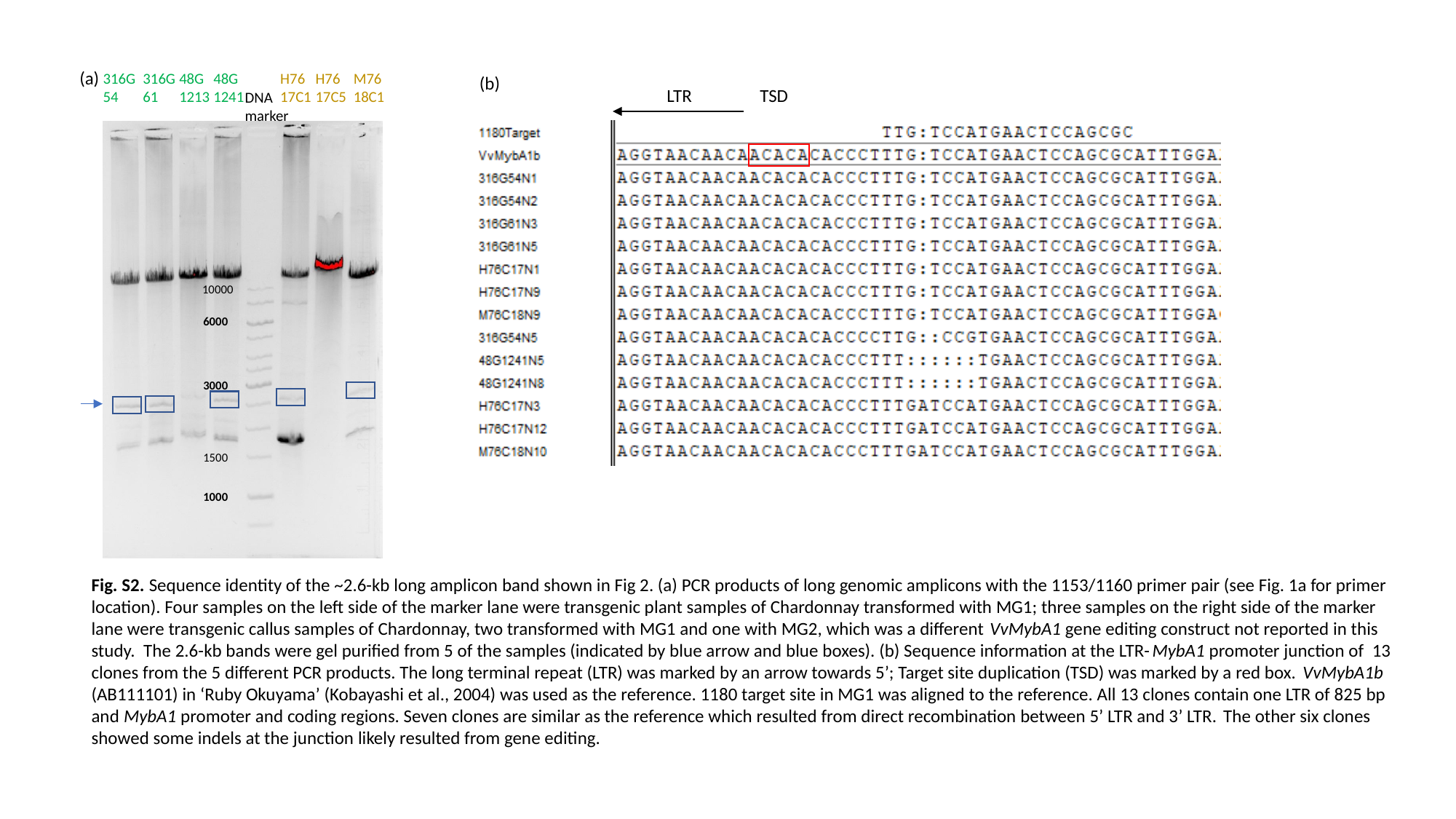

(a)
316G54
316G61
48G 1213
48G 1241
H76 17C1
H76 17C5
M76 18C1
DNA marker
10000
6000
3000
1500
1000
(b)
LTR
TSD
Fig. S2. Sequence identity of the ~2.6-kb long amplicon band shown in Fig 2. (a) PCR products of long genomic amplicons with the 1153/1160 primer pair (see Fig. 1a for primer location). Four samples on the left side of the marker lane were transgenic plant samples of Chardonnay transformed with MG1; three samples on the right side of the marker lane were transgenic callus samples of Chardonnay, two transformed with MG1 and one with MG2, which was a different VvMybA1 gene editing construct not reported in this study. The 2.6-kb bands were gel purified from 5 of the samples (indicated by blue arrow and blue boxes). (b) Sequence information at the LTR-MybA1 promoter junction of 13 clones from the 5 different PCR products. The long terminal repeat (LTR) was marked by an arrow towards 5’; Target site duplication (TSD) was marked by a red box. VvMybA1b (AB111101) in ‘Ruby Okuyama’ (Kobayashi et al., 2004) was used as the reference. 1180 target site in MG1 was aligned to the reference. All 13 clones contain one LTR of 825 bp and MybA1 promoter and coding regions. Seven clones are similar as the reference which resulted from direct recombination between 5’ LTR and 3’ LTR. The other six clones showed some indels at the junction likely resulted from gene editing.

## Slide 3
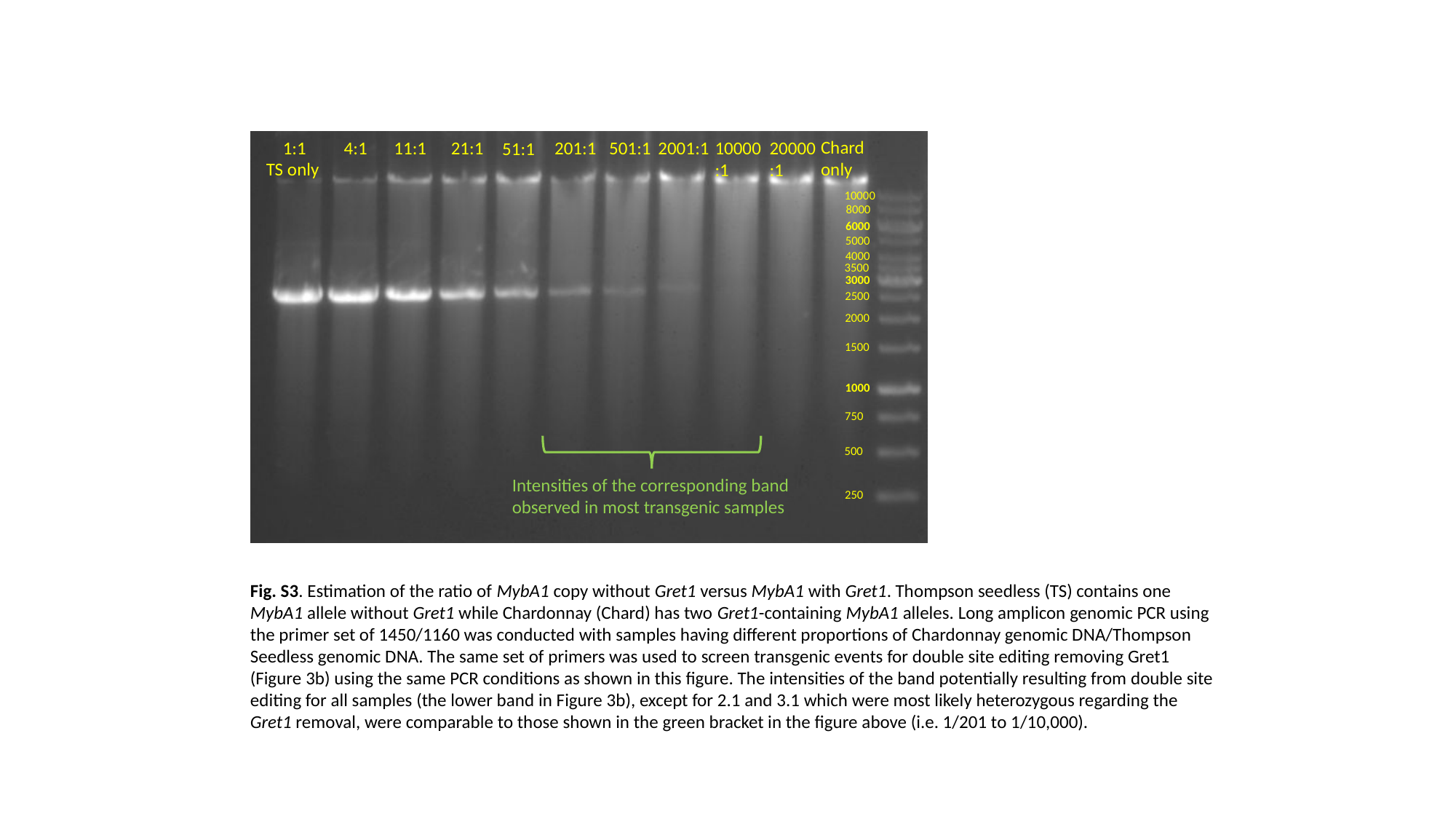

Chard
only
1:1
4:1
11:1
21:1
201:1
501:1
2001:1
10000
:1
20000
:1
51:1
TS only
10000
8000
6000
5000
4000
3500
3000
2500
2000
1500
1000
750
500
Intensities of the corresponding band observed in most transgenic samples
250
Fig. S3. Estimation of the ratio of MybA1 copy without Gret1 versus MybA1 with Gret1. Thompson seedless (TS) contains one MybA1 allele without Gret1 while Chardonnay (Chard) has two Gret1-containing MybA1 alleles. Long amplicon genomic PCR using the primer set of 1450/1160 was conducted with samples having different proportions of Chardonnay genomic DNA/Thompson Seedless genomic DNA. The same set of primers was used to screen transgenic events for double site editing removing Gret1 (Figure 3b) using the same PCR conditions as shown in this figure. The intensities of the band potentially resulting from double site editing for all samples (the lower band in Figure 3b), except for 2.1 and 3.1 which were most likely heterozygous regarding the Gret1 removal, were comparable to those shown in the green bracket in the figure above (i.e. 1/201 to 1/10,000).

## Slide 4
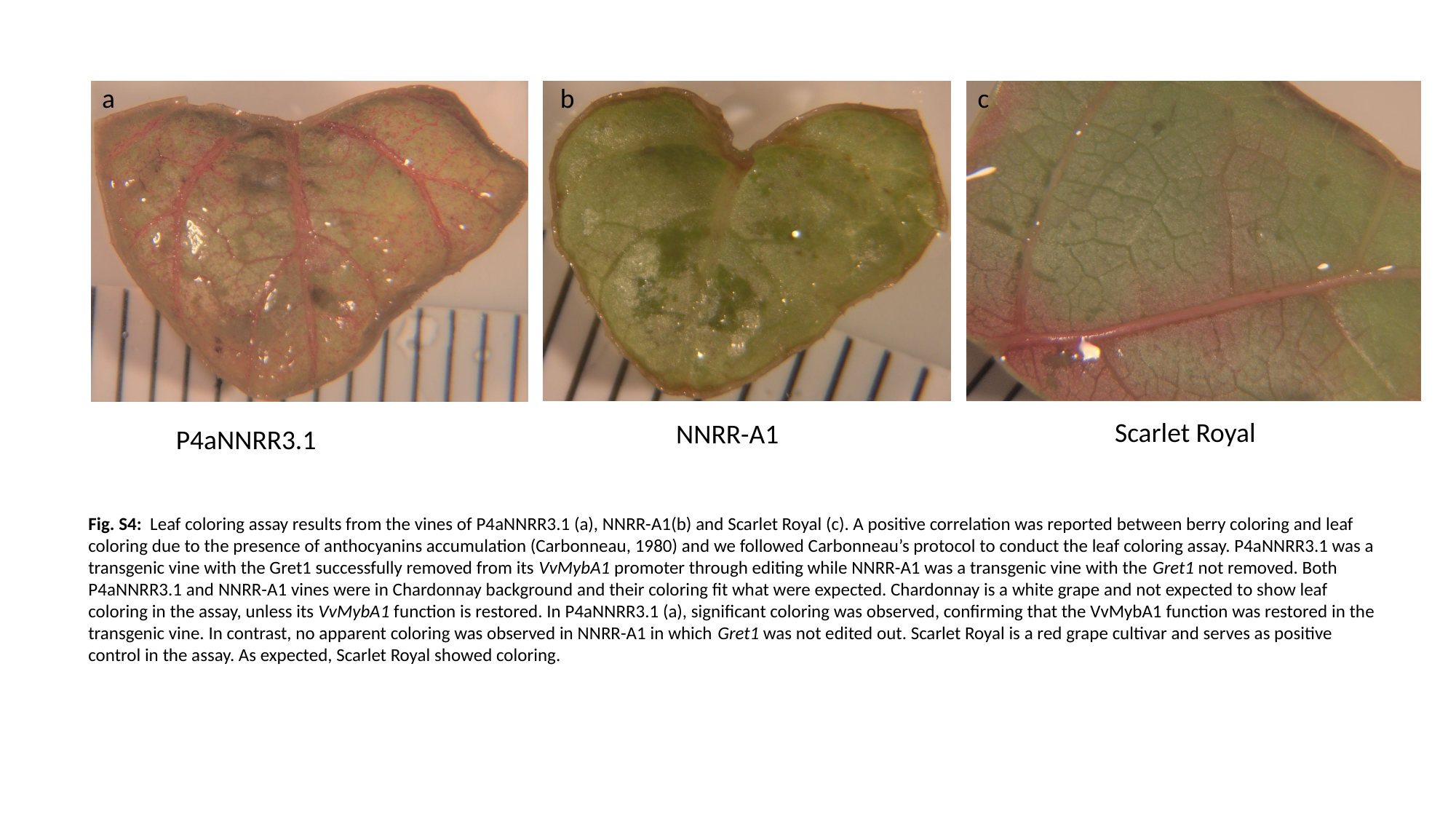

a
b
c
Scarlet Royal
NNRR-A1
P4aNNRR3.1
Fig. S4: Leaf coloring assay results from the vines of P4aNNRR3.1 (a), NNRR-A1(b) and Scarlet Royal (c). A positive correlation was reported between berry coloring and leaf coloring due to the presence of anthocyanins accumulation (Carbonneau, 1980) and we followed Carbonneau’s protocol to conduct the leaf coloring assay. P4aNNRR3.1 was a transgenic vine with the Gret1 successfully removed from its VvMybA1 promoter through editing while NNRR-A1 was a transgenic vine with the Gret1 not removed. Both P4aNNRR3.1 and NNRR-A1 vines were in Chardonnay background and their coloring fit what were expected. Chardonnay is a white grape and not expected to show leaf coloring in the assay, unless its VvMybA1 function is restored. In P4aNNRR3.1 (a), significant coloring was observed, confirming that the VvMybA1 function was restored in the transgenic vine. In contrast, no apparent coloring was observed in NNRR-A1 in which Gret1 was not edited out. Scarlet Royal is a red grape cultivar and serves as positive control in the assay. As expected, Scarlet Royal showed coloring.
